# A Cipher Based on Prefix Codes

**DOI:** 10.3390/s21186236

**Published:** 2021-09-17

**Authors:** Otokar Grošek, Viliam Hromada, Peter Horák

**Affiliations:** 1 Faculty of Electrical Engineering and Information Technology, Slovak University of Technology in Bratislava, Ilkovičova 3, 812 19 Bratislava, Slovakia; otokar.grosek@stuba.sk; 2 School of Interdisciplinary Arts & Sciences, University of Washington, Tacoma, WA 98402, USA; horak@uw.edu

**Keywords:** cryptography, prefix-codes, prefix-codes-based cryptosystem

## Abstract

A prefix code, a *P*-code, is a code where no codeword is a prefix of another codeword. In this paper, a symmetric cipher based on prefix codes is proposed. The simplicity of the design makes this cipher usable for Internet of Things applications. Our goal is to investigate the security of this cipher. A detailed analysis of the fundamental properties of *P*-codes shows that the keyspace of the cipher is too large to mount a brute-force attack. Specifically, in this regard we will find bounds on the number of minimal *P*-codes containing a binary word given in advance. Furthermore, the statistical attack is difficult to mount on such cryptosystem due to the attacker’s lack of information about the actual words used in the substitution mapping. The results of a statistical analysis of possible keys are also presented. It turns out that the distribution of the number of minimal *P*-codes over all binary words of a fixed length is Gaussian.

## 1. Introduction

A prefix code, a *P*-code, is a code where no codeword is a prefix of another codeword [1]. *P*-codes have been used in symmetric cryptography for a long time [2,3].

The oldest known example of a *P*-code is the Argenti code [2] (16th century). *P*-codes were also used by Peter the Great, where the plaintext was the Cyrillic alphabet [2]. Furthermore, the Soviet cipher known as VIC [3], used *P*-codes as one of the rounds during the encryption. Finally, in the mid of the 20th century, the properties of *P*-codes were studied by the leading scholars in the area, including Shannon, Fano, Huffman, etc. Nowadays, one area of cryptography which employs prefix codes is the DNA cryptography [4], in which many cryptosystems utilize binary prefix codes as the plaintext space [5,6,7,8].

**Example** **1.** *To illustrate the usage of P-codes for encryption, let us consider Table 1, where each lowercase letter is mapped to a codeword of a binary prefix code; each codeword is of length four or five. It is readily seen that no codeword is a prefix of any other codeword. The plaintext, written as a sequence of lowercase letters, is encrypted by substituting each letter with the corresponding binary string and concatenating the strings into the resulting ciphertext.*

*Let the message be iotuser. Substituting each letter by its corresponding binary codeword, the encrypted form is 11101111100111010010010110110001. The table is secret and known only to the sender and receiver, therefore only they are able to uniquely decipher the encrypted message, by using the property of prefix codes that no codeword is a prefix of any other codeword. The attacker, having observed the ciphertext, must solve two things to find the secret key, i.e., the used mapping: first of all, he/she must divide the message correctly into the variable-length segments and then he/she must find the correct substitution.*

The plaintext alphabet may contain special symbols called null-ciphers, which are inserted into the plaintext during encryption and when decrypted are represented by the empty string. The usage of null-ciphers makes the frequency analysis of the ciphertext more difficult.

The memory complexity of the cipher depends on the size of the table, i.e., on the size of the plaintext alphabet and the corresponding codewords. For example, let the plaintext alphabet be the ASCII alphabet (128 symbols) and each variable-length codeword be approximately 10-bit long. Then the size of the table would be roughly 1280 bits, e.g., 160 bytes.

Due to the simplistic design of the cryptosystem and its low memory requirements, it is usable as a symmetric cipher for IoT applications. Securing the communication of IoT devices is currently extensively studied [9,10,11], with one notable proposal of an encryption algorithm using prefix codes as its plaintext alphabet [12]. The corresponding key-exchange of the symmetric key can be performed by algorithms dedicated for IoT, e.g., the Merkle-tree authenticated KEM [13].

The paper is organized as follows. The proposal of a cryptosystem based on *P*-codes is presented in Section 2. In Section 3, we collect fundamental properties of *P*-codes. Section 4 provides an algorithm for finding the set of all minimal dictionaries of *P*-codes with respect to a given string *x*. Statistical data obtained by running this algorithm for all strings of length up to 26 are presented in Section 5. Finally, the preliminary cryptanalysis of the cryptosystem is described in Section 6.

## 2. The Proposal of a Cryptosystem Based on P-Codes

To be able to propose a cipher based on prefix codes, we first recall the definition of a prefix code.

**Definition** **1.** *Let A be an alphabet and P be a set, $P\subset {\left\{0,1\right\}}^{+}$. Then a code is a bijection κ:A→P; elements of P are called codewords and P is also called a dictionary of the code. Specifically, a prefix code, for short a P-code, is a code where no codeword is a prefix of another codeword. A message x is a concatenation of finitely many words from the dictionary P.*

In what follows, the dictionary *P* will consist of binary words, while the alphabet A is the English A-Z alphabet, the ASCII alphabet, etc. For a set of binary words *P*, $P^{+}$ stands for the set of all finite concatenations of elements of *P*.

We now state the definition of the cryptosystem based on *P*-codes.

**Definition** **2.** *Let A be a plaintext alphabet, let P be a set of prefix codes P. Then a symmetric substitution cryptosystem based on P-codes is a five-tuple (P,C,K,E,D) where:*

1. *The plaintext alphabet P=A.*
2. *The ciphertext alphabet C={0,1}.*
3. *The keyspace K consists of two-tuples K=(P,κ), where P∈P and κ is a bijection A→P.*
4. *For each K=(P,κ)∈K, the encryption rule $e_{K}\in E$, $e_{K}=\kappa :A\to P$.*
5. *For each K=(P,κ)∈K, the decryption rule $d_{K}\in D$, $d_{K}=\kappa^{-1}:P\to A$.*

The property $d_{K}\left(e_{K}\left(m\right)\right)=m$ follows directly from 4 and 5. If *m* is a string of plaintext symbols, $m\in P^{+}$, $m=m_{1}m_{2}\ldots m_{n}$, then as usual, its encryption is the sequence $x=x_{1}x_{2}\ldots x_{n}=e_{K}\left(m_{1}\right)e_{K}\left(m_{2}\right)\ldots e_{K}\left(m_{n}\right)=\kappa \left(m_{1}\right)\kappa \left(m_{2}\right)\ldots \kappa \left(m_{n}\right)\in C^{+}$. Decryption is done in the same manner. We note that this algorithm is a substitution cipher, in which it is difficult to distinguish respective encrypted plaintext symbols in the ciphertext.

The encryption can be performed as a simple look-up in the look-up table of the used *P*-code. Since each plaintext character is mapped independently of the other symbols, the encryption resembles the so-called ECB-mode of block ciphers. However, to further enhance the security of the cipher, special symbols called null-ciphers can be employed. These special symbols are also part of the plaintext alphabet and have their corresponding images under the mapping κ. They are randomly inserted into the plaintext and thus get encrypted as some binary strings. However, during the decryption, they are represented as empty strings and therefore do not change the meaning of the original plaintext.

The decryption is performed by identifying codewords in the ciphertext and mapping them to their preimages under the mapping κ. The recognition of codewords can be easily implemented with the usage of a finite state automaton, which would process the input ciphertext on a bit-by-bit basis and have separate accepting states for each codeword. On reaching one of these accepting states, the automaton would recognize the corresponding codeword, return to the initial state and process the ciphertext further.

### Key Generation

The generation of a random key K=(P,κ) consists of two steps:

1. Generate the set of codewords *P*.
2. Generate the mapping κ.

If we want to generate a random set of codewords *P* for an alphabet, say with 128 characters, we have several possibilities. One is to generate a random string *x* such that there is a huge number of dictionaries having cardinality |V|=128, where *x* is the concatenation of all codewords of the dictionary, in some order. Then, one would try to find one of the dictionaries by using Algorithm 1 presented in Section 2. The shortest possible length of such a string *x* which would have a dictionary with 128 codewords is |x|≥7×128=896. Unfortunately, such a string cannot be effectively processed by this algorithm, since the algorithm exhaustively searches all possible partitions of the string, which takes an exponential time in the length of *x*.

Another approach is to generate a random binary string *x* of a sufficient length, e.g., in the case |V|=128, |x|≥896 and then try to generate a random partition of *x* and test, whether the resulting partition of *x* forms a *P*-code. If not, one may try to generate another random partition of *x* and test it again. However, this approach might also take a lot of time if the length of *x* is not sufficiently large. For example, if we want to find a *P*-code *V* such that |V|=128, the least possible length of *x* is 896. In this case, the only suitable partition is the partition of *x* into 128 7-bit substrings. However, the number of all partitions of a 896-bit string into 128 substrings is 895127≈2523. Another disadvantage of this method is the fact that some of the substrings might be the same, therefore the resulting *P*-code would be of a smaller cardinality than 128.

A more efficient way is to directly generate a set of random integers $n_{1},n_{2},\ldots ,n_{128}$ and test if these numbers satisfy the well-known Kraft inequality ([14,15]):

(1) $$\sum_{i=1}^{128}2^{-n_{i}}\leq 1.$$

If so, then there exists a *P*-code with codewords of lengths $n_{i},i=1,2,\ldots ,128$, and there are many ways how to construct it. For example, one may construct a binary tree with 128 leaves, where *i*-th leaf is at $n_{i}$ level under the root. Then, each leaf directly represents a codeword of length $n_{i}$ of some *P*-code with 128 codewords. This process can further be randomised during the binary tree’s construction, which leads to a randomized algorithm that generates different *P*-codes even for the same sequence of lengths $n_{i}$. Straightforward implementation of this approach on a portable computer is able to generate a random *P*-code with 128 codewords in approx. 20–30 ms.
**Algorithm 1:** Algorithm FindVx(x,T) for finding all minimal *P*-codes that decode *x*
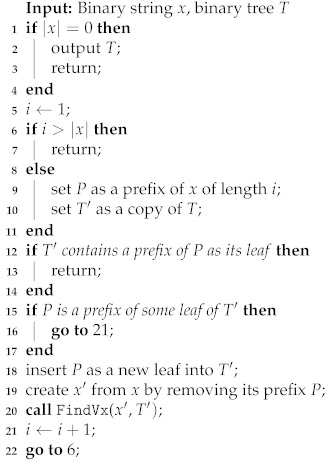


Once such a set of codewords *P* has been generated, the generation of the mapping κ can be done by generating a random bijection A→P. Due to implementational simplicity, this mapping may be also fixed in a sense that the first symbol of alphabet A will always be mapped to the first codeword of *P*, the second symbol of A will be mapped to the second codeword of *P*, etc. Thus, the certification authority (CA) distributing the keys among the IoT devices needs to only distribute the set of codewords *P* and not the mapping κ itself.

The efficiency of the cipher can be measured, as is common, by a transmission rate. Suppose that the alphabet A comprises all 8-bit characters, i.e., |A|=256. Generally, the average codeword length is $\bar{n}=\sum n_{i}p_{i}$, where $p_{i}$ is the probability of occurrence of character $a_{i}\in A$, $n_{i}$ is the length of the corresponding codeword of $a_{i}$. Then, $\bar{n}$ is expected to be more than that given by some efficient (e.g., Huffman) coding. Thus, in this case, the efficiency can be measured as the fraction $\frac{\bar{n}}{8}$, i.e., the average number of bits of ciphertext per one encrypted character. Further, $\frac{\bar{n}}{8}>1$ and the higher this fraction is, the more *P*-codes are available for this string. For security reasons, our coding does not adhere to the obvious rule used in efficient coding, where $p_{i}<p_{j}$ implies $n_{i}>n_{j}$, since we do not want to give the attacker any additional information about the used *P*-code.

## 3. Set of Minimal Dictionaries with Respect to a Given String

If the dictionary *P* is known, the decision whether the string *x* was written using this dictionary is trivial. However, if the dictionary is not known, which represents the attacker’s situation, the attacker must consider all possible dictionaries, which could have been used to create *x*. The key ingredient in the study of all possible dictionaries is the notion of a minimal dictionary with respect to the known *x*.

**Definition** **3.** *Let x be a binary string. Then a set V such that $x\in V^{+}$ is called minimal with respect to x, if for any w∈V, $x\notin {\left(V-w\right)}^{+}$. The collection of all minimal sets V, $x\in V^{+}$, is denoted by $V_{x}$.*

**Example** **2.** *Given a string x of length |x|>1, there are at least two minimal sets containing x, $|V_{x}|\geq 2$, say $V_{1}=\left\{x\right\}$ and $V_{2}=\left\{0,1\right\}$ with an exception when x consists of only zeroes or ones, then $V_{2}=\left\{0\right\}$ or $V_{2}=\left\{1\right\}$, respectively.*

**Theorem** **1.** *Let x be a string. Then a set $V\subset {\left\{0,1\right\}}^{+}$ is minimal with respect to x if and only if V is a partition of x into substrings.*

As *V* is a set, then each substring occurs in *V* once; *V* is not a multiset, repetitions do not count. Since *V* is a partition of *x*, we have that $x\in V^{+}$.

**Proof.**  (⇐). Let elements of *V* form a partition of *x* into substrings; say $x=v_{1}v_{2}\ldots v_{t}$, where $v_{i}$’s are not necessarily different. Assume, by contradiction, that *V* is not minimal. Then there would be w∈V such that $x\in {\left(V-w\right)}^{+}$. This in turn implies that $x=v_{j_{1}}\ldots v_{j_{t-1}}$. If $|v_{j_{1}}\left|>\right|v_{1}|$ or $|v_{j_{1}}\left|<\right|v_{1}|$, then $v_{j_{1}}$ would be a prefix of $v_{1}$ or vice versa. Therefore, $|v_{j_{1}}\left|=\right|v_{1}|$ which implies $v_{j_{1}}=v_{1}$. The rest of the proof is done by induction on the length of *x*.

(⇒) If *V* is minimal with respect to *x*, then $x\in V^{+}$. Therefore, *V* has to contain elements whose concatenation is *x*; these elements form a partition of *x* into substrings. Because of minimality of *V*, there is no other element there. □

In order to show that it is computationally infeasible to break our cipher by brute-force, we will investigate the number of *P*-codes containing a piece of ciphertext *x*. In fact, it suffices to show that the number of minimal *P*-codes containing *x*, the subset of all *P*-codes, is large.

All minimal *P*-codes can be obtained by partitioning *x* into substrings (Theorem 1) and then checking, whether this partitioning satisfies the prefix property.

For a given string *x* of length ν, the number of different partitions into substrings is $2^{\nu -1}$, as each delimiter is placed in between two consecutive symbols.

The following example illustrates this procedure.

**Example** **3.** *If x=0011, then there exist 8 partitions. For each partition, we will verify whether the obtained set satisfies the prefix property. We will have following dictionaries:*

- *if there is no delimiter, then the corresponding P-code is {0011}*
- *one delimiter:*
*partition 0|011 leads to V={0,011}, which does not satisfy the prefix property,*
*partition 00|11 results in a P-code {00,11},*
*001|1 implies a P-code {001,1}.*
- *two delimiters:*
*0|0|11, leads to a P-code {0,11},*
*0|01|1 does not satisfy the prefix property,*
*00|1|1 we get a P-code {00,1}*
- *3 delimiters: 0|0|1|1 leads a P-code {0,1}.*

*Thus, for x=0011, the procedure leads to 6 minimal P-codes, i.e., $|V_{x}|=6$. Later, we will see that for x long enough, $|V_{x}|\ll 2^{|x|-1}$.*

The structure of dictionaries from $V_{x}$, is presented in the following theorem.

**Theorem** **2.** *Let x be a binary string of length ν and C, D be two dictionaries in $V_{x}$. Then:*

*(1)*  *There are no dictionaries C⊂D, where C is a proper subset of D.*
*(3)*  *There are no dictionaries C≠D such that C∩D is a dictionary.*
*(3)*  *There are no dictionaries C≠D such that C∪D is a dictionary.*

**Proof.**  (1). Let *x* be a binary string $x=x_{1}x_{2}x_{3}\ldots x_{\nu }$. Suppose that *C* and *D* are two dictionaries such that $C,D\in V_{x}$ and C⊂D, say $C=\left\{c_{1},\ldots ,c_{s}\right\},D=\left\{d_{1},\ldots ,d_{s},d_{s+1},\ldots d_{t}\right\}$. Without loss of generality, let $c_{1}=d_{1},c_{2}=d_{2},\ldots ,c_{s}=d_{s}$. According to Theorem 1, *D* comprises substrings of *x*, therefore there is an element $d_{i}\in D$ such that

$$x=\underset{d_{i}}{\underset{︸}{x_{1}x_{2}\ldots x_{k}}}x_{k+1}\ldots x_{\nu }.$$

Similarly, there is $c_{j}\in C$ such that

$$x=\underset{c_{j}}{\underset{︸}{x_{1}x_{2}\ldots x_{m}}}x_{m+1}\ldots x_{\nu }.$$

We distinguish 3 cases:

$$1.\;m<k\;x=\underset{c_{j}}{\underset{︸}{x_{1}x_{2}\ldots x_{m}}}\ldots x_{k}\ldots x_{\nu },$$

$$2.\;m>k\;x=\underset{c_{j}}{\underset{︸}{x_{1}x_{2}\ldots x_{k}\ldots x_{m}}}\ldots x_{\nu },$$

$$3.\;m=k\;x=\underset{c_{j}}{\underset{︸}{x_{1}x_{2}\ldots x_{k=m}}}\ldots x_{\nu }.$$

By assumption, $c_{j}=d_{j}$, which in turn implies that the codeword $d_{j}$ is a prefix of $d_{i}$, a contradiction. Analogously, $d_{i}=c_{i}$, which means that $c_{i}$ is a prefix of $c_{j}$.

Therefore, m=k. If we remove the prefix $x_{1}x_{2}\ldots x_{k=m}$ from the string *x*, the above procedure can be repeated on the shorter string $x_{k+1}\ldots x_{\nu }$. Now, the induction on ν finishes the proof.

Item (2) follows directly from (1). If there were two different dictionaries *C* and *D* of *P*-codes such that C∩D is a dictionary as well, then it would be true that there are two different dictionaries, C∩D and *C*, for which C∩D⊊C, contradicting (1).

Item (3) follows from (1) as well. If C∪D were a *P*-code, then C⊊C∪D, contradicting (1). □

Now we turn our attention to the cardinality of $V_{x}$. The following theorem and corollary deal with the lower bound. The upper bound will be estimated statistically in Section 5.

We recall that the function τ(n) counting the number of divisors of *n*, including 1 and *n*, can be easily computed from the prime factorization of *n*: If $n=p_{1}^{\alpha_{1}}\ldots p_{r}^{\alpha_{r}}$, then $\tau \left(n\right)=\left(\alpha_{1}+1\right)\ldots \left(\alpha_{r}+1\right)$ [16].

In the following, we use the common notation $1^{\nu }={\underset{︸}{11\ldots .11}}_{\nu -\mathrm{times}}$ and $0^{\nu }={\underset{︸}{00\ldots .00}}_{\nu -\mathrm{times}}$. We also use the notation $\bar{x}$ to represent the binary complement of *x*.

**Theorem** **3.** *Let $x=1^{\nu }$ and τ(ν) be the function which counts number of divisors of ν. Then $|V_{x}|=\tau \left(\nu \right)$. Similarly, for $\bar{x}=0^{\nu }$, $|V_{\bar{x}}|=\tau \left(\nu \right)$.*

**Proof.**  It is readily seen that a dictionary $V\in V_{x}$ contains only one element. Otherwise, for any two elements in *V* one would be a prefix of the other, a contradiction. Hence $V=\{1^{d}\}$ and because $x=1^{d}1^{d}\ldots 1^{d}$, d|ν and vice versa. The proof follows. The case of $\bar{x}=0^{\nu }$ is analogous. □

**Proposition** **1.** *Let x be a string and $\bar{x}$ be its binary complement. $V=\{v_{1},v_{2},\ldots ,v_{n}\}$$\in V_{x}$ is a dictionary of the string x, if and only if, $\bar{V}=\left\{\bar{v_{1}},\bar{v_{2}},\ldots ,\bar{v_{n}}\right\}$ is a dictionary of the string $\bar{x}$, i.e., $\bar{V}\in V_{\bar{x}}$.*

**Proof.**  Let $x=v_{i_{1}}v_{i_{2}}\ldots$, $v_{i_{j}}\in V$. Then $\bar{x}=\bar{v_{i_{1}}}\;\bar{v_{i_{2}}}\ldots$, $\bar{v_{i_{j}}}\in \bar{V}$ and vice versa. The proof follows. □

**Corollary** **1.** *For each string x of length ν, $|V_{x}|\geq \tau \left(\nu \right)$. The equality is attained only for $x=1^{\nu }$, $x=0^{\nu }$, and also for all strings with ν=2. For all other strings $|V_{x}|>\tau \left(\nu \right)$.*

**Proof.**  The first part of the statement follows from Theorem 3 and from $|V_{x}|=\tau \left(2\right)=2$ for all strings of length 2. To finish the proof we show that for all other strings $|V_{x}|>\tau \left(\nu \right)$. We recall that now $x\neq 0^{\nu },x\neq 1^{\nu }$.

1. Let *d* be a divisor of ν. If we partition $x=v_{1}v_{2}\ldots v_{t}$, where $|v_{i}|=d,t=\frac{\nu }{d}$, then the set $V=\{v_{1},\ldots ,v_{t}\}$ is a dictionary of the string *x*, since the words $v_{i}$ are of the same length and therefore cannot be prefixes of each other. In this way, we can find τ(ν) different dictionaries of the string *x*. Therefore $|V_{x}|\geq \tau \left(\nu \right)$. We note that the above argument works also in the case ν is a prime.
2a. Let $x=1^{r}w$, r≥1, where the leftmost digit of *w* is 0, with the exception of the string $x=1^{\nu -1}0$. Then {1,w} is also a dictionary of the string *x*. Moreover, |w|>1, therefore we have found a dictionary with codewords of different lengths, i.e., a dictionary not included in the previous case. In the case $x=1^{\nu -1}0$ we can consider a dictionary $\left\{1^{\nu -1},0\right\}$ and have once again found a dictionary with codewords of different lengths, as ν−1≥2. Therefore $|V_{x}|>\tau \left(\nu \right)$.
2b. Let $x=0^{r}w$, where the leftmost digit of *w* is 1. Due to Proposition 1$|V_{x}\left|=\right|V_{\bar{x}}|$ and we can consider the complement $\bar{x}$ of *x* and continue as in the previous case.

This proves our corollary. □

## 4. Algorithm for Finding the Set $V_{x}$

In this section, we describe an algorithm for finding the set $V_{x}$. This algorithm was employed for all strings of lengths up to 26.

For a given binary string *x* of length ν, the algorithm finds all such minimal *P*-codes *V* that $x\in V^{+}$. Since each *P*-code can be viewed as a binary tree *T* whose leaves represent the codewords of the *P*-code, the algorithm recursively searches all $2^{\nu -1}$ divisions of the string *x* into partitions and checks whether these substrings may form the leaves of some binary tree *T*. If so, then the algorithm outputs the tree *T* that also represents such *P*-code *V*, where $x\in V^{+}$.

The input of the recursive algorithm is a binary string *x*, whose prefixes are potential codewords and a binary tree *T* with already-found codewords. In the beginning, the algorithm is used with the original string *x* and a binary tree *T* with only one node—its root.

Steps 5–20 take a prefix *P* of *x* of lengths 1 up to |x| and check whether it may be a codeword of some P-code by keeping up a binary tree *T* of already identified codewords. The following situations might arise for a prefix *P* of the string *x* and a binary tree *T* (its *working copy*$T^{\prime }$):

**Steps 12–14** If one of the leaves of $T^{\prime }$ is a prefix of *P*, then it is also a prefix of *x*. Therefore the current call of the algorithm terminates, since it is not possible to create another codeword from the current string *x*.
**Steps 15–17** If the string *P* is a prefix of some leaf of $T^{\prime }$, i.e., a prefix of some codeword, then this prefix is ignored and a prefix of length +1 is considered (i.e., the jump to the step 21).
**Steps 18–20** The string *P* might be a codeword of some *P*-code. Therefore, it is inserted to the tree $T^{\prime }$ as a new leaf and the algorithm is recursively called with the rest of *x* after removal of *P* (i.e., the string $x^{\prime }$ in the step 19) and with the tree $T^{\prime }$.

For example, if we apply Algorithm 1 to a string x=01010, we find the following *P*-codes:


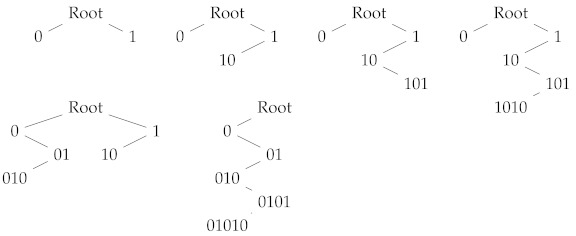


The trees represent the following *P*-codes, respectively:

{0,1},{0,10},{0,101},{0,1010},{010,10},{01010}.

## 5. Statistical Approach for Cardinality of the Set $V_{x}$

**Figure 1:**
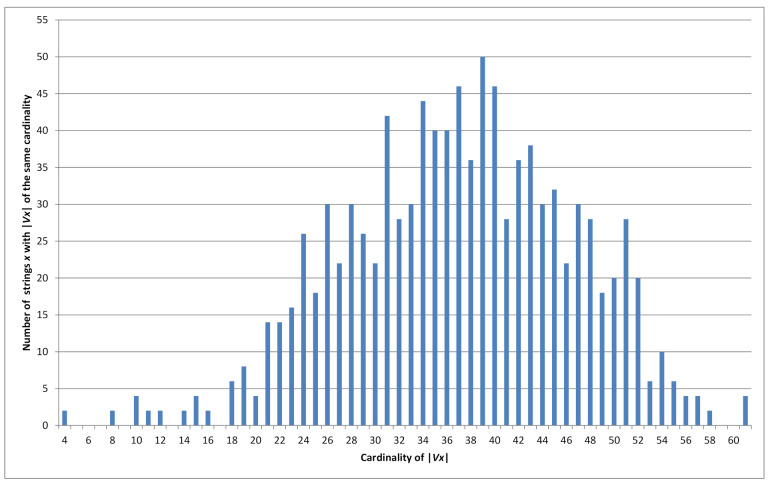
Cardinality distribution histogram for ν=10.

Consider all $2^{10}$ binary strings of length ν=10. For each string, we find the set $V_{x}$ and its cardinality. Cardinality distribution histogram of $|V_{x}|$ is in Figure 1. For example, 50 strings of length ν=10 have the property that 39 *P*-codes can be constructed from them, i.e., for 50 strings $x\in {\left\{0,1\right\}}^{10}$ it is true that $|V_{x}|=39$. Maximum cardinality $|V_{x}|=61$ is reached by four strings *x* (last value of the histogram).

We have estimated the upper bound for $\max_{x\in {\left\{0,1\right\}}^{\nu }}\left|V_{x}\right|=h\left(\nu \right)=h$ experimentally by using the log-linear regression to be $2^{0.5122\nu +0.5253}$. As mentioned above, this number is less than $2^{\nu -1}$. To do this, we collected the data which are included in Table 2. Description of its columns are in the next subsection.

### Results of Our Exhaustive Search ν=1,…,26

We implemented Algorithm 1 in programming language C++ and used it to calculate the cardinalities of sets $V_{x}$ for all possible binary strings *x* of lengths ν=1,2,…,26. For each value of ν∈{1,2,…,26}, we generated all $2^{\nu }$ strings *x* of length ν and determined all possible *P*-codes, i.e., the set $V_{x}$, and $|V_{x}|$ for each possible string *x*. Our algorithm checked $2^{\nu }2^{\nu -1}=2^{2\nu -1}$ instances for each ν∈{1,2,…,26}. As a consequence we were able to generate histograms for distribution of cardinalities of $V_{x}$. One such example is presented in Figure 1.

We used computational resources available at the HPC center at the Slovak University of Technology in Bratislava, where the computation used 72 computational nodes and took 1 day 20 h and 53 min, i.e., it performed at a rate approx. 416 *x* strings of length 26 per second.

At this rate, the calculation would take approx. 3 days for ν=27, one week for ν=28, two weeks for ν=29 and approx. one year and three months for ν=34. However, in reality the periods would be even longer, since the rate would slow down, because of longer strings *x*.

We present the results in Table 2, with the following columns:

1. ν—The length of the binary string *x*, ν=1,2,…,26
2. *k*—Upper bound on |V|
3. μ—Estimated mean μ for $|V_{x}|$
4. *s*—Estimated standard deviation *s* for $|V_{x}|$
5. 5—Estimated maximum of the function $2^{\nu }f_{N}$, where $f_{N}$ is the Gaussian density function $N(\mu ,s^{2})$, i.e., $\frac{2^{\nu }}{\sqrt{2\pi s^{2}}}$. In other words, the theoretical number of strings $x\in {\left\{0,1\right\}}^{\nu }$ with their value of $|V_{x}|$ equal to the estimated mean value μ.
6. 6—The measured number of strings $x\in {\left\{0,1\right\}}^{\nu }$ with their value of $|V_{x}|$ equal to the most probable value of $|V_{x}|$.
7. $Min=min\{|V_{x}|\}$—The smallest number of *P*-codes for a string $x\in {\left\{0,1\right\}}^{\nu }$, i.e., the smallest $|V_{x}|$. Due to Corollary 1 it is equal τ(ν).
8. $Max=max\{|V_{x}|\}$—The largest number of *P*-codes for a string $x\in {\left\{0,1\right\}}^{\nu }$, i.e., the largest $|V_{x}|$.
9. *h*—The estimated upper bound h(ν) using log-linear regression from the collected data.

**Figure 2:**
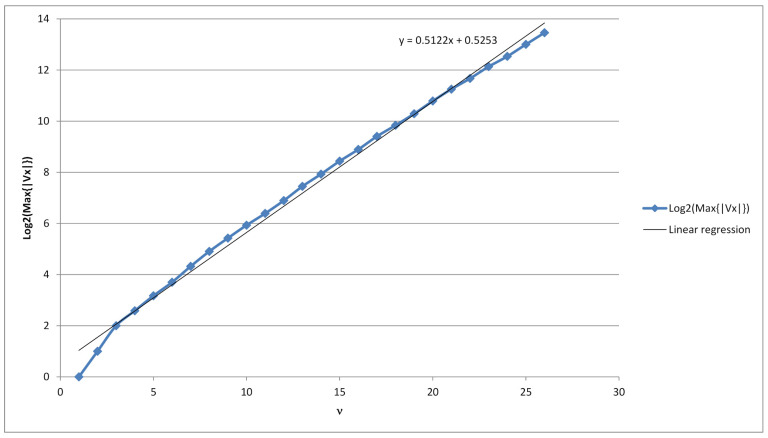
Linear regression for $\log_{2}Max\left\{V_{x}\right\}$, ν=1…,26.

Comparing columns 5 and 6 one can see how sharp is our estimation for the most probable value. From this it follows that this estimation is acceptable. Comparing the last two columns it follows that the discrepancy between our estimation for h=h(ν) and the true value is also acceptable. These columns also display the range of occurrences for $|V_{x}|$. (see also Figure 2).

Let *x* be a binary string of the length ν and $V_{x}$ be the set of all *P*-codes that can be obtained from the string *x* by Algorithm 1. Moreover, let $h=\max_{x\in {\left\{0,1\right\}}^{\nu }}\left|V_{x}\right|$. Then for each t=1,2,…,h and $x\in {\left\{0,1\right\}}^{\nu }$, we define the characteristic function

(2) $$\chi_{t}\left(x\right)=\left\{\begin{array}{cc} 1, & if|V_{x}|=t;\\ 0, & \mathrm{otherwise} \end{array}\right.$$

Let $X_{\nu ,t}$ be a random variable

(3) $$X_{\nu ,t}=\sum_{x\in {\left\{0,1\right\}}^{\nu }}\chi_{t}\left(x\right),$$

defined on the sample space Ω={1,2,…,h}. For instance, if ν=10,t=39 we have $X_{10,39}=50$ (see graph in Figure 1). Moreover, because of the complement property mentioned above we have $|V_{x}\left|=\right|V_{\bar{x}}|$, and $X_{\nu ,t}$ is an even number for ν>1.

Next, we need a variant of the Central limit theorem [17].

**Lemma** **1.** *Let a density functions $f_{\nu ,t}\left(x\right)$ of independent random variables $X_{\nu ,t}$ be bounded by a constant h and their characteristic function is non negative. Then densities of random variables $Y_{n}=\frac{\sum_{t=1}^{n}X_{\nu ,t}}{s\sqrt{n}}$ converge to the density of Gaussian random variable.*

**Figure 3:**
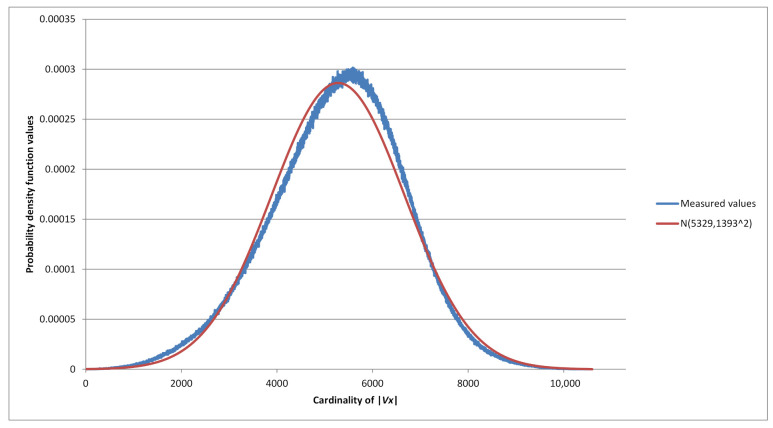
The estimate of the density function of a random variable $X_{26,t}$.

**Figure 4:**
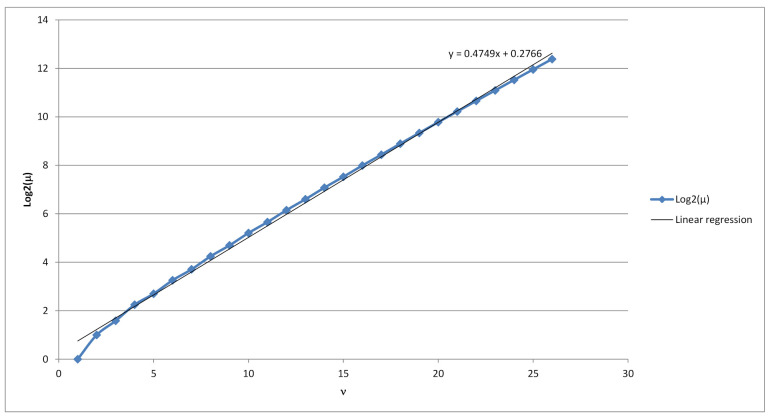
Linear regression for $\log_{2}\mu$, ν=1…,26.

**Figure 5:**
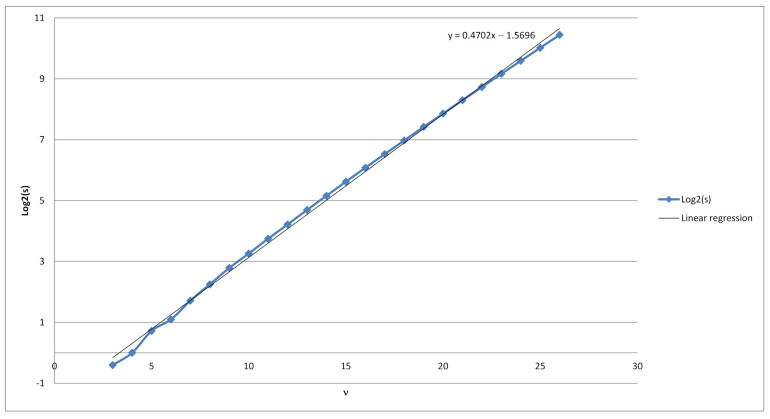
Linear regression for $\log_{2}s$, ν=3…,26.

Hence our random variable $X_{\nu ,t}$ possesses Gaussian probability distribution with a density function $f_{\nu ,t}$. Using exhaustive search for values ν=1,2,…,26 subsequent linear regression revealed that the random variable $X_{\nu ,t}$ has a slightly biased normal distribution (see Figure 3) with the mean value of $\mu =2^{0.4749\nu +0.2766}$ (see Figure 4) and a standard deviation of $s=2^{0.4702\nu -1.5696}$ (see Figure 5). From there we get an estimate on the number of strings *x* of length ν for which $|V_{x}|$ is equal to the most probable value, i.e., the mean of random variable $X_{\nu ,t}$:

$$\max\{|\{x\in {\left\{0,1\right\}}^{\nu }:|V_{x}|=t\}|\}\approx$$

(4) $$2^{\nu }\sqrt{2\pi s^{2}}=2^{\nu }\sqrt{2\pi }\times 2^{0.4702\nu -1.5696}=\sqrt{2\pi }\times 2^{1.4702\nu -1.5696}.$$

## 6. Preliminary Cryptanalysis of the Proposed Cipher Based on P-Codes

The security of the proposed cryptosystem is based on the fact that there exist a rather large (potentially infinite if the length of codewords is not upper-bounded) number of dictionaries for plaintext alphabets. As mentioned earlier, the cryptanalysis can be further made more complicated by employing null-ciphers into encryption, i.e., the code *P* may contain several codewords that will be decoded as an empty string. We recommend to insert a random number of these null-ciphers into the beginning and end of the ciphertext and also to randomly insert them into ciphertext during the encryption as well. In our opinion, this security measure substitutes the usage of encryption modes, since the encryption without null-ciphers behaves similarly to ECB-mode, where two equal sequences of plaintext characters would be encrypted as two equal binary sequences. With the addition of randomly inserted null-ciphers, this is no longer true.

We also suppose that each message has its own encryption key, i.e., the used *P*-code.

Let us consider the ciphertext-only attack, i.e., the attacker has observed a ciphertext *x*. The brute-force attack on the cryptosystem would consist of the exhaustive search for the used key (P,κ). To investigate the security of the system under this attack, in this paper we study the properties of *P*-codes to show that the keyspace is too large to mount such an attack.

The size of keyspace depends on both the size of alphabet A and on the size of P which is determined by fundamental properties of *P*-codes.

As can be seen on Figure 4, the average number of minimal dictionaries with respect to a given string grows exponentially with regards to the length of the binary string. Therefore, one approach to have a complexity of $2^{128}$ of finding the correct minimal dictionary, is to let the length of the ciphertext to be at least 128<0.4749ν+0.2766, i.e., 268<ν. In this case, if the ciphertext has at least 269 bits, then there exist on average at least $2^{128}$ possible dictionaries, which could have been used to generate such a ciphertext. Thus, even if the attacker tries to find the correct dictionary by searching through all possible minimal dictionaries with respect to the ciphertext, he/she has to search through $2^{128}$ dictionaries.

## 7. Conclusions

In this paper, a symmetric cipher based on prefix codes has been proposed. Our main goal was to investigate the security of the cipher regarding the amount of information about the key, i.e., the used random *P*-code, that is available to the attacker by observing a ciphertext, i.e., a string of concatenated codewords of the *P*-code. The encryption algorithm and methods how to generate such random *P*-code are presented in Section 2. In Section 3 we defined a notion of a minimal dictionary of a *P*-code with respect to a given string and we identified the minimal bound on the number of all dictionaries with respect to a given string. An exponential algorithm which finds all minimal dictionaries with respect to a given string is proposed in Section 4. Statistical approach for finding the upper bound on the number of all minimal dictionaries with respect to a given string is presented in Section 5. Our analysis shows that the number of possible *P*-codes derived from a string increases exponentially with the length of the corresponding string, therefore the keyspace of the cipher increases exponentially as well, making the brute-force attack difficult. In Section 6, we present a preliminary cryptanalysis of the proposed cipher. In order to retain the simplicity of the cipher and to prevent some basic attacks, e.g., the statistical analysis of the ciphertext, we suggest to use the null-ciphers during the encryption.

Some technicalities about possible coders and decoders for *P*-codes can be further found in [18]. Information on effective decoding algorithms can be found in [19,20] and on memory-efficient representation of prefix codes can be found in [21].

Future plans include finding the sharp lower bound of the cardinality of a dictionary with respect to a given string *x*.

## Figures and Tables

**Table 1:** *P*-code example.

a	b	c	d	e	f	g	h	i	j
00000	00001	00010	00011	1101	00101	00110	00111	1110	01001
k	l	m	n	o	p	q	r	s	t
01010	01011	01100	01101	1111	01111	10000	10001	10010	10011
u	v	w	x	y	z				
10100	10101	10110	10111	11000	11001				

**Table 2:** Results of our exhaustive search ν=1,2,…,26. (see the description of columns above.)

ν	*k*	μ	*s*	5	6	Min	Max	*h*
1	1	1	0	-	2	1	1	2.05
2	2	2	0	-	4	2	2	2.93
3	2	3	1	4.22	4	2	4	4.18
4	2	4.75	1.0000	6.38	6	3	6	5.95
5	3	6.50	1.6461	7.76	10	2	9	8.49
6	3	9.56	2.1372	11.95	18	4	13	12.11
7	3	13.06	3.2814	15.56	22	2	20	17.28
8	4	18.95	4.7508	21.50	30	4	30	24.64
9	4	25.94	6.9036	29.59	40	3	43	35.14
10	4	36.91	9.5817	42.64	50	4	61	50.12
11	4	50.45	13.4382	60.80	74	2	84	71.48
12	5	70.89	18.5844	87.93	118	6	119	101.95
13	5	96.92	25.8554	126.40	162	2	175	145.40
14	5	134.88	35.6985	183.10	218	4	244	207.38
15	5	184.21	49.2433	265.47	326	4	347	295.76
16	6	254.16	67.5362	387.13	528	5	476	421.82
17	6	346.25	92.3633	566.14	688	2	678	601.62
18	6	474.43	125.8496	830.99	988	6	916	858.04
19	6	644.47	171.1880	1221.82	1414	2	1253	1223.76
20	7	877.99	232.1493	1801.95	1990	6	1771	1745.35
21	7	1189.26	314.4021	2661.06	2876	4	2440	2489.26
22	7	1612.52	424.7709	3939.26	4264	4	3262	3550.24
23	7	2177.56	573.1570	5838.84	6252	2	4497	5063.43
24	8	2940.84	771.6567	8673.73	9240	8	5935	7221.59
25	8	3959.67	1037.8313	12,898.32	13,716	3	8215	10,299.60
26	8	5328.85	1393.0635	19,218.48	20,242	4	11,276	14,689.52

## Data Availability

The data presented in this study and the source code of the algorithm are available on request from the corresponding author.
